# The recent advances of mast cells in the pathogenesis of atopic dermatitis

**DOI:** 10.3389/falgy.2025.1668742

**Published:** 2025-09-30

**Authors:** Zhenzhen Xiao, Yunqian Zhuo, Rui Li, Yingjian Tan

**Affiliations:** 1 Department of Dermatology, Fuzhou First General Hospital, Fuzhou, China; 2 Department of Dermatology, Venereology and Allergology, Charité-Universitätsmedizin Berlin, Berlin, Germany

**Keywords:** atopic dermatitis, mast cells, FcϵRI, MRGPRX2, c-Kit

## Abstract

Mast cells play a critical role in the pathogenesis of atopic dermatitis (AD), a chronic inflammatory skin disease characterized by itch, eczema, and barrier dysfunction. These immune cells are abundant in the skin and are activated in response to allergens, irritants, and microbial products. Upon activation, mast cells release a variety of mediators, including histamine, proteases, cytokines, and chemokines, which contribute to the inflammation and pruritus observed in AD. Recent studies have highlighted the importance of mast cell-derived IL-4, IL-13, and IL-31 in promoting Th2-type immune responses and itch sensation. Moreover, interactions between mast cells and sensory neurons may further exacerbate neuroimmune inflammation. Mast cells also influence skin barrier integrity by modulating keratinocyte function and disrupting tight junctions. Their numbers and activation state are often elevated in AD lesions, correlating with disease severity. Targeting mast cell activation or blocking their mediators has shown promise in preclinical models, offering potential therapeutic strategies. Overall, mast cells are increasingly recognized as key contributors to the initiation and amplification of AD, making them an important focus for understanding disease mechanisms and developing new treatments.

## 1 Introduction

Atopic dermatitis (AD) is a chronic, relapsing inflammatory skin disease characterized by intense pruritus, dry skin, and eczematous lesions. It commonly begins in childhood but can persist or develop in adulthood (1). The pathogenesis of AD is complex and involves a combination of genetic predisposition, skin barrier dysfunction, environmental factors, and immune dysregulation (2). A key feature of AD is the involvement of the immune system, particularly type 2 immune response, which includes increased activity of T helper 2 (Th2) cells and elevated levels of cytokines such as IL-4, IL-13, and IL-31 (3). These immune changes contribute to inflammation, itch, and impaired skin barrier function (4, 5). Among the immune cells involved in AD, mast cells play a critical and multifaceted role. Traditionally recognized for their involvement in allergic responses, mast cells are abundant in the skin and are strategically located near blood vessels and nerve endings (6, 7). They can be activated through both IgE-dependent and non-IgE pathways, releasing a wide range of mediators such as histamine, cytokines, and proteases (8). These mediators contribute to pruritus, inflammation, and skin remodeling in AD. Due to their central position in skin immunity and inflammation, mast cells are an important focus in understanding AD pathogenesis and developing new therapeutic strategies.

**Figure 1:**
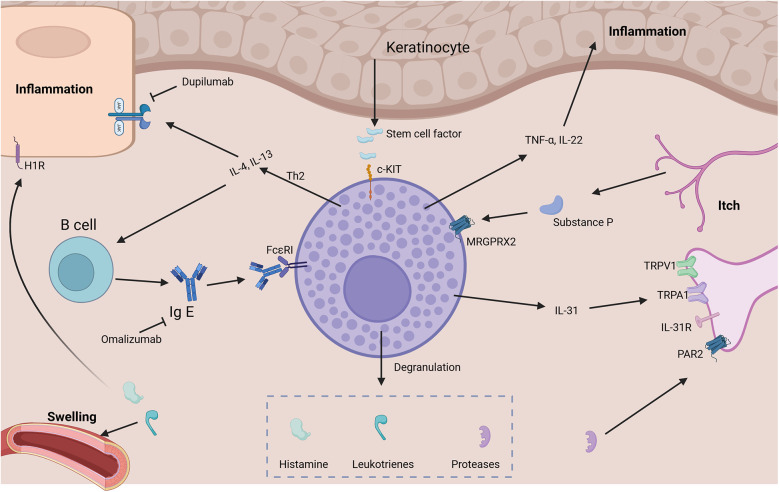
Schematic showing mast cell–driven pathways in inflammation and itch. Key elements include IgE/FcϵRI and MRGPRX2-mediated degranulation, release of cytokines (IL-4, IL-13, IL-31), histamine, leukotrienes, and proteases. Keratinocytes and receptors such as TRPV1 participate in itch signaling, while therapeutic antibodies like Dupilumab and Omalizumab are indicated as modulators.

Mast cells are tissue-resident immune cells derived from hematopoietic progenitors, widely distributed throughout the human body. They are particularly abundant in areas exposed to the external environment, such as the skin, respiratory tract, gastrointestinal mucosa, and around blood vessels and nerves (9). These strategic locations allow mast cells to serve as first responders to environmental stimuli, including pathogens, allergens, and physical injury. Functionally, mast cells play a central role in innate and adaptive immunity (Figure 1). They contain cytoplasmic granules rich in histamine, heparin, proteases (such as tryptase and chymase), and various cytokines (10). Upon activation, they rapidly release these mediators, initiating inflammatory responses. In addition to their role in allergic reactions, mast cells contribute to host defense against pathogens, wound healing, angiogenesis, and tissue remodeling. They can also modulate the function of other immune cells, including T cells, B cells, eosinophils, and dendritic cells, thereby influencing both local and systemic immunity. Mast cells express a variety of surface receptors that regulate their activation, survival, and function. The KIT receptor (also known as c-Kit), which binds stem cell factor (SCF), is crucial for mast cell development, survival, and activation (11). Another key receptor is the high-affinity IgE receptor (FcεRI), essential for allergic responses. Pattern recognition receptors such as Toll-like receptors (TLRs) detect pathogens. Chemokine receptors (e.g., CCR3, CXCR4) mediate cell migration. MrgprB2, the murine homolog of human MRGPRX2, mediates non-IgE-dependent pseudo-allergic responses (12). Together, these receptors enable mast cells to play key roles in immunity and inflammation.

In the skin, mast cells are predominantly located in the dermis, especially near blood vessels, lymphatics, hair follicles, and peripheral nerves. Their role in the skin extends beyond traditional allergy responses. They participate in maintaining skin homeostasis, regulating vascular permeability, and facilitating communication between immune and non-immune cells. One of their primary roles in the skin is to mediate inflammatory responses. On activation, skin mast cells release preformed mediators such as histamine and tryptase, which contribute to vasodilation, edema, and recruitment of additional immune cells to the site of injury or infection. They also secrete newly synthesized cytokines like TNF-α, IL-4, IL-13, and IL-31, which are key players in the development of chronic skin inflammation and pruritus, particularly in disorders like AD and urticaria (13, 14). Furthermore, mast cells in the skin interact closely with sensory nerves, forming part of a neuroimmune circuit that contributes to itch and pain. For example, IL-31 released by mast cells can directly stimulate sensory neurons, triggering itch. Histamine, one of the most well-known mast cell mediators, also activates H1 and H4 receptors on sensory nerves to provoke itching. Mast cells also influence skin barrier integrity. They regulate keratinocyte proliferation and differentiation and can alter the expression of tight junction proteins, which are crucial for maintaining an effective barrier against environmental insults. In inflammatory skin diseases, overactivation of mast cells can lead to barrier disruption and sustained inflammation, creating a cycle that perpetuates disease progression. In summary, mast cells are multifunctional immune cells widely distributed throughout the body, with particularly important roles in the skin. In addition to their well-established function in allergic reactions, they contribute to host defense, tissue repair, neuroimmune signaling, and skin barrier maintenance. Their dysregulation or hyperactivation in the skin can lead to chronic inflammatory and allergic skin conditions, making them a critical focus in dermatological research and therapy.

Mast cell degranulation is a key process by which these immune cells exert their effects during immune responses, particularly in allergic and inflammatory conditions. Degranulation refers to the rapid release of pre-stored bioactive mediators from secretory granules within mast cells into the surrounding tissue (15). This process is typically triggered by the cross-linking of IgE antibodies bound to the high-affinity FcεRI receptors on the mast cell surface upon allergen exposure. However, other stimuli such as complement components (e.g., C3a, C5a), neuropeptides, physical injury, and certain microbial products can also induce degranulation. There are three main types of mast cell degranulation: classical (anaphylactic) degranulation, piecemeal degranulation, and cytolysis. Classical degranulation involves the rapid fusion of granule membranes with the plasma membrane, leading to the explosive release of granule contents like histamine, tryptase, heparin, and proteases (16). This is the most well-known form and is typically associated with immediate hypersensitivity reactions. Piecemeal degranulation is a slower, selective release of specific granule contents without complete granule-emptying, often observed during chronic inflammation. Cytolysis involves the release of granule contents due to mast cell death, which may occur in response to certain toxins or pathogens. The mediators released during degranulation play diverse roles. Histamine increases vascular permeability and smooth muscle contraction, leading to redness, swelling, and itching. Proteases such as tryptase contribute to tissue remodeling and recruit other immune cells. Heparin acts as an anticoagulant, and cytokines like TNF-α amplify the inflammatory response.

Mast cell degranulation is tightly regulated, as uncontrolled or excessive release of mediators can result in tissue damage and contribute to the pathogenesis of allergic diseases such as asthma, AD, and anaphylaxis. Understanding the mechanisms underlying degranulation has led to the development of targeted therapies, including mast cell stabilizers and receptor antagonists, which aim to control allergic and inflammatory symptoms by preventing or reducing mediator release.

## 2 FcεRI-mediated IgE-dependent activation pathway

The FcεRI-mediated IgE-dependent activation pathway plays a central role in the pathogenesis of AD, a chronic, recurrent inflammatory skin disorder characterized by intense pruritus, skin barrier dysfunction, and immune dysregulation (17). FcεRI is a key surface receptor expressed on mast cells, basophils, and, to a lesser extent, on dendritic cells and eosinophils. It binds IgE with high affinity, and its activation initiates a cascade of immune responses upon allergen exposure.

FcεRI^−/−^ mice are not sensitive to stimuli in the AD model, which suggests a FcεRI-mediated induction of the IgE-dependent activation pathway that induces mast cell activation and is involved in the pathogenesis of AD. In individuals with AD, serum IgE levels are often elevated, and specific IgE antibodies are produced against various environmental allergens (e.g., dust mites, pollens, microbes) (18). Previous studies have confirmed high serum IgE titer is associated with high disease activity, with IgE communication in areas of AD lesions overbinds to FcεRI on the surface of mast cells and basophils, induce effector cell activation degranulation. Upon subsequent exposure, allergens cross-link adjacent IgE molecules on FcεRI, leading to mast cell activation and degranulation.

This activation triggers the immediate release of preformed mediators such as histamine, tryptase, and heparin, followed by the production of lipid mediators (e.g., prostaglandins and leukotrienes) and the synthesis of type 2 inflammation. Histamine and IL-31 induce pruritus by stimulating sensory nerves. IL-4 and IL-13 promote a Th2-skewed immune response, suppress antimicrobial peptide production, and impair skin barrier function by downregulating filaggrin and other structural proteins. TNF-α and other cytokines recruit eosinophils, dendritic cells, and T cells to the skin, amplifying chronic inflammation (19).

In addition to mast cells, FcεRI expression on Langerhans cells (a type of dendritic cell in the skin) enables allergen uptake and presentation to naïve T cells, further promoting Th2 polarization. This reinforces the allergic inflammation loop in AD. The overactivation of the FcεRI-IgE pathway is also associated with disease severity. Patients with more severe forms of AD tend to have higher levels of allergen-specific IgE and increased FcεRI expression on immune cells. Consequently, targeting this pathway has become a therapeutic strategy. For example, omalizumab, a monoclonal antibody that binds to free IgE and prevents its interaction with FcεRI, has shown efficacy in reducing symptoms in some AD patients (20). In summary, the FcεRI-mediated IgE-dependent activation pathway is a pivotal mechanism driving allergic inflammation, itch, and skin barrier dysfunction in AD. It links environmental allergens to immune cell activation and cytokine production, making it a key target for therapeutic intervention in allergic skin diseases.

## 3 MRGPRX2/B2-mediated IgE-independent activation pathway

The Mas-related G protein-coupled receptor X2 (MRGPRX2 in humans; MrgpxB2 in mice) mediates a non-IgE-dependent activation pathway of mast cells and has recently gained attention for its role in the pathogenesis of AD (21). Unlike the classical IgE-FcεRI pathway, which requires allergen-specific IgE for mast cell activation, the MRGPRX2 pathway can be triggered directly by various cationic ligands, including neuropeptides (e.g., substance P), antimicrobial peptides (e.g., LL-37), drugs, and host-derived peptides released during inflammation or tissue damage (21). In the context of AD, MRGPRX2-mediated mast cell activation contributes to inflammation and pruritus through alternative mechanisms that do not require prior sensitization. This pathway is especially important in chronic or non-atopic forms of AD, where elevated IgE levels may not be present. Activation of MRGPRX2 leads to rapid mast cell degranulation and the release of histamine, proteases, and pro-inflammatory cytokines, which contribute to itch, skin inflammation, and barrier disruption (22).

One of the hallmark roles of MRGPRX2 in AD is its involvement in neuroimmune interactions. Neuropeptides such as substance P, released from sensory nerves during stress or mechanical stimulation, can bind to MRGPRX2 on mast cells and trigger degranulation. This leads to a feed-forward loop of inflammation and itch: mast cell mediators stimulate nerve endings, leading to further neuropeptide release and continued activation of mast cells. This loop may underlie the chronic itch characteristic of AD and is particularly relevant in the “itch-scratch” cycle that worsens the disease. Additionally, the expression of MRGPRX2 is upregulated in the lesional skin of AD patients, and its activation is associated with heightened mast cell responsiveness. Increased MRGPRX2 signaling may also contribute to the skin's hypersensitivity to environmental triggers, such as irritants or mechanical stress, further exacerbating the condition (23). The expression of protease-activated receptor-2 (PAR-2) is upregulated, and this receptor can be activated by tryptase released from mast cells, mediating histamine-independent itch. This suggests that the “tryptase–PAR-2 axis” plays a significant role in the pruritus associated with atopic dermatitis (24). Therapeutically, targeting MRGPRX2 offers a promising strategy for treating AD, particularly in patients who do not respond well to IgE-targeted therapies. Inhibitors that block MRGPRX2 activation or its downstream signaling pathways could help reduce mast cell-mediated inflammation and pruritus without affecting the beneficial roles of IgE in host defense (25, 26). In summary, the MRGPRX2-mediated, non-IgE-dependent activation pathway represents an important mechanism in AD pathogenesis. It contributes to mast cell-driven inflammation and itch, particularly in IgE-independent or chronic forms of the disease. Understanding and targeting this pathway could open new avenues for more effective and personalized therapies for AD.

## 4 The cytokines sereted by mast cells

Mast cells are key effector cells in allergic and inflammatory responses, and their role in AD is largely mediated through the secretion of various cytokines (27). These cytokines significantly influence the development, persistence, and severity of AD by promoting inflammation, disrupting skin barrier function, and amplifying pruritus. Upon activation—through either IgE-dependent (via FcεRI) or non-IgE-dependent (e.g., MRGPRX2) pathways—mast cells rapidly release a variety of pro-inflammatory cytokines, including IL-4, IL-5, IL-6, IL-13, IL-31, and TNF-α (Table 1). These mediators play diverse and crucial roles in AD pathogenesis (28).

**Table 1:** Key mediators released during mast cell degranulation are listed with their major physiological functions and specific roles in atopic dermatitis. These include cytokines, chemokines, histamine, proteases, and lipid-derived factors. Collectively, they promote inflammation, itch, barrier dysfunction, and chronic disease progression.

Mediator	Primary function	Roles in atopic dermatitis
Histamine	Potent vasodilator; increases vascular permeability; induces pruritus via H1 and H4 receptors;	Promotes intense itching and erythema; contributes to neurogenic inflammation and scratch–itch cycle in AD (78);
Tryptase	Serine protease involved in extracellular matrix remodeling and activation of protease-activated receptor-2;	Enhances keratinocyte cytokine release, disrupts skin barrier, and amplifies inflammation (26);
PGD₂	Lipid mediator regulating vascular tone and leukocyte recruitment;	Attracts Th2 cells and eosinophils via CRTH2 receptor, facilitating type 2 inflammation in AD (23);
IL-5	Promotes eosinophil growth, activation, and survival;	Drives eosinophilic inflammation in AD lesions, enhancing tissue damage and pruritus (45);
IL-6	Pro-inflammatory cytokine with pleiotropic effects on immune cell activation and differentiation;	Contributes to acute inflammation and amplifies cytokine cascade in AD (32);
IL-4/IL-13	Central type 2 cytokine involved in IgE class switching and barrier dysfunction;	Suppresses skin barrier proteins and antimicrobial peptides, driving chronic type 2 inflammation (8, 29);
IL-31	Key pruritogenic cytokine activating sensory neurons;	Directly triggers itch and promotes nerve fiber elongation in AD skin, linking immune and neural responses (38);
TNF-α	Pro-inflammatory cytokine activating endothelial cells and leukocytes;	Amplifies inflammatory cell recruitment, enhances cytokine release, and exacerbates skin damage (35);

IL-4 and IL-13 are central to the type 2 immune response characteristic of AD. They promote the differentiation of naïve T cells into Th2 cells, enhance IgE production by B cells, and impair skin barrier integrity by downregulating key structural proteins such as filaggrin (29, 30). These effects contribute to increased skin permeability and heightened sensitivity to environmental allergens and microbes. Studies have shown that the number of scratches in mice with conditional knockout of dorsal root ganglia IL-4R in mice with a significant reduction in AD models (31). IL-5 is important for the recruitment and activation of eosinophils, which are often elevated in AD and contribute to tissue damage and chronic inflammation. IL-6 plays a role in acute phase responses and further amplifies inflammation by enhancing the activation of T cells and other immune cells. It may also contribute to skin barrier dysfunction by affecting keratinocyte differentiation (32). IL-31, known as a “pruritogenic” cytokine, is particularly important in the context of itch. It directly activates TRPV1 and TRPA1 channels on the surface of sensory neurons, leading to the sensation of itch, which is a hallmark of AD (31, 33). The release of IL-31 by mast cells contributes to the vicious itch-scratch cycle, which exacerbates skin damage and inflammation (34). TNF-α is a potent pro-inflammatory cytokine that promotes the expression of adhesion molecules on endothelial cells, facilitating the recruitment of leukocytes to the skin. It also stimulates other immune and non-immune cells to produce additional inflammatory mediators, thus sustaining the inflammatory environment in AD lesions (35).

Collectively, these cytokines orchestrate a complex network of immune responses that maintain and intensify the disease. Targeting mast cell-derived cytokines has become an important therapeutic strategy in AD. Biologics such as dupilumab, which blocks IL-4 and IL-13 signaling, have demonstrated significant efficacy in reducing symptoms and improving skin barrier function. In summary, mast cell-derived cytokines are pivotal in the pathogenesis of AD. Through their diverse effects on immune modulation, pruritus induction, and barrier disruption, these mediators contribute to both the acute flare-ups and the chronicity of the disease. Understanding their roles provides valuable insights into the development of targeted treatments for AD.

## 5 Pruritus induced by mast cells through neuroimmune mechanisms

Pruritus is one of the hallmark symptoms of AD and other allergic conditions. Mast cells, traditionally known for their role in immune responses and allergy, are also key contributors to pruritus through neuroimmune mechanisms (36). This type of itch arises when mast cells interact with the nervous system, leading to the activation of sensory nerve fibers that transmit the sensation of itching. Mast cells are abundant in the skin, particularly in areas affected by AD, where they are activated by various stimuli, including allergens, irritants, and inflammatory mediators. These mediators not only contribute to inflammation but also directly influence sensory neurons in the skin, which are responsible for transmitting itch signals to the brain (37).

Histamine, a well-known mediator released from mast cells, binds to H1 receptors on sensory neurons in the skin. This interaction directly triggers the sensation of itch by activating the itch-specific C-fibers. Histamine is one of the first and most potent mediators involved in mast cell-induced pruritus. IL-31, a cytokine secreted by Th2 cells and mast cells, has been identified as a major pruritogenic mediator. It binds to the IL-31 receptor on sensory neurons, leading to itch sensation. Elevated levels of IL-31 in AD patients are correlated with increased itch severity, making it a key target for therapeutic intervention. Mast cells release neuropeptides, such as substance P and calcitonin gene-related peptide (CGRP), which act on nearby sensory nerve endings to enhance itching (38). These neuropeptides also contribute to the neurogenic inflammation in AD by promoting vasodilation and recruiting more inflammatory cells to the site of activation. The interaction between mast cells and sensory neurons is bidirectional. Sensory nerves can release neurotransmitters such as substance P that activate mast cells, leading to further degranulation and amplification of the inflammatory response. This creates a vicious cycle of mast cell activation and itch, exacerbating the condition (39). Recent studies have elucidated a neuro-immune crosstalk between sensory neurons and mast cells in AD. For instance, scratching-induced inflammation requires activation of pain-sensing nociceptors, which in turn trigger mast cell degranulation, linking neurogenic signaling to allergic inflammation (40). This neuro-mast cell axis represents a novel mechanism by which mechanical stimuli and neural signals exacerbate AD pathology, providing additional targets for intervention.

Targeting mast cell-mediated pruritus through neuroimmune mechanisms offers a promising strategy for treating chronic itch in AD (41). IL-31 receptor antagonists, H1 antihistamines, and substance P blockers are potential therapeutic approaches being explored in clinical trials. These therapies aim to break the itch-scratch cycle, providing significant relief to patients suffering from severe pruritus. In conclusion, mast cells mediate pruritus in AD through complex interactions with the nervous system (42). By releasing histamine, cytokines like IL-31, and neuropeptides, mast cells activate sensory neurons and contribute to the persistent itch seen in AD. Targeting these pathways may offer new therapeutic options for managing pruritus in patients with AD and other pruritic conditions (43).

## 6 Interactions with other immune cells

Mast cells are not only effector cells but also central regulators of the immune network in AD, engaging in extensive crosstalk with various immune cell types. They can influence dendritic cell maturation and function, promoting the activation and polarization of T cells toward a Th2 phenotype, which is critical for the type 2 inflammatory response characteristic of AD (44). Mast cells also interact with T cells directly, releasing cytokines such as IL-4, IL-5, and IL-13 that enhance Th2 differentiation and maintain chronic inflammation (45).

In addition, mast cells recruit and activate eosinophils through the release of chemokines and cytokines, further amplifying tissue inflammation and contributing to pruritus and barrier dysfunction (46). Cross-talk with B cells can promote IgE production, establishing a feedback loop that heightens mast cell activation via FcεRI (47). Recent evidence also indicates interactions with innate lymphoid cell (ILC), particularly ILC2s, which further drive type 2 cytokine responses in the skin (43).

These multifaceted interactions highlight mast cells as orchestrators of both innate and adaptive immunity in AD. By modulating other immune cell functions, mast cells not only contribute directly to inflammation and pruritus but also shape the broader immune microenvironment, sustaining chronic disease and influencing treatment responses. Recognizing these complex cellular networks provides a more comprehensive understanding of AD pathophysiology and may reveal novel therapeutic targets.

## 7 Mast cells-related Ad mouse model

Animal models have been instrumental in advancing our understanding of mast cell-related mechanisms in AD (48). These models allow for controlled investigation of the roles of specific immune cells, cytokines, and genetic factors in the pathogenesis of AD. Among them, mast cell–related models have provided key insights into how mast cells contribute to skin inflammation, pruritus, and barrier dysfunction in AD.

### 7.1 Nc/Nga mouse model

The Nc/Nga mouse is one of the most widely used spontaneous models of AD. Under conventional housing conditions, these mice develop AD-like skin lesions with characteristics similar to human disease, including infiltration of mast cells, increased serum IgE, and Th2 cytokine expression (49). Mast cell degranulation and histamine release are prominent features. This model has been used to test antihistamines, mast cell stabilizers, and immunomodulators, confirming the involvement of mast cells in both inflammation and pruritus (50).

### 7.2 Ovalbumin (OVA)-induced model

In this antigen-induced model, mice are sensitized and challenged with ovalbumin (OVA), a protein that acts as an allergen (51, 52). Repeated exposure leads to skin inflammation, Th2 immune responses, and mast cell accumulation and activation in the dermis. This model mimics allergic sensitization and highlights the role of IgE–FcεRI–mast cell signaling in AD-like pathology (53).

### 7.3 Calcipotriol (Mc903)-induced model

Topical application of calcipotriol (MC903), a vitamin D3 analog, induces AD-like skin lesions in mice (54). This model replicates epidermal barrier disruption, type 2 inflammation, and itch, and is associated with mast cell infiltration and activation. It is particularly useful for studying non-IgE pathways and the neuroimmune mechanisms of pruritus, including the role of IL-31 and MrgprX2-related mast cell activation (55).

### 7.4 Mast cell–deficient mice

Genetically modified mouse strains lacking mast cells, such as Kit^W−sh/W−sh^ mouse, have been used to directly assess the function of mast cells in AD. When subjected to AD-like conditions (e.g., allergen challenge or MC903 application), these mice exhibit reduced skin inflammation and pruritus compared to wild-type controls (56). Reconstitution of mast cells in these mice restores the disease phenotype, further confirming the critical role of mast cells. More recently, newer mast cell–deficient models that avoid off-target effects of c-Kit mutations (e.g., Cpa3-Cre; Mcl-1^fl/fl^) have provided cleaner insights into mast cell-specific contributions to AD without affecting other immune cells (57).

### 7.5 MrgprB2-dependent itch models

Mouse models using MrgprB2 agonists such as compound 48/80 or neuropeptides like substance P can selectively activate mast cells via non-IgE pathways (58). These models are used to study neuroimmune interactions and mast cell–induced pruritus. They help dissect alternative mechanisms of mast cell activation relevant to AD, especially in patients who do not exhibit high IgE levels. Mast cell–related AD models, ranging from spontaneous to induced and genetically modified systems, have been essential in unraveling the complex role of mast cells in AD (59). These models have not only enhanced our understanding of the disease but also provided platforms to evaluate mast cell–targeted therapies, including inhibitors of FcεRI, MrgprX2, IL-31, and histamine pathways.

## 8 Mast cell-related treatments towards AD

Targeting mast cell-related pathways offers a promising therapeutic approach for AD, given their central role in inflammation, pruritus, and skin barrier dysfunction. Therapies such as omalizumab, an anti-IgE antibody, reduce IgE-FcεRI-mediated mast cell activation and are beneficial in some AD patients with elevated IgE. Dupilumab, targeting IL-4Rα, effectively blocks IL-4 and IL-13 signaling, improving inflammation and skin barrier function. Nemolizumab, which blocks the IL-31 receptor, is particularly effective for relieving chronic itch. Novel strategies targeting MRGPRX2, a receptor involved in non-IgE-dependent mast cell activation, are under investigation and may benefit patients with chronic or IgE-independent AD. Mast cell stabilizers (e.g., cromolyn sodium) and antihistamines provide symptomatic relief, though with limited efficacy. Additionally, JAK inhibitors such as upadacitinib and abrocitinib block cytokine signaling downstream of mast cell activation and show strong anti-inflammatory and antipruritic effects. Overall, mast cell-targeted therapies, by disrupting key inflammatory pathways, offer diverse and effective options for managing AD, particularly in moderate-to-severe or treatment-resistant cases.

### 8.1 Anti IgE-FcεRI pathway

Omalizumab is a humanized monoclonal antibody that targets IgE. It binds to free circulating IgE, preventing its interaction with FcεRI on the surface of mast cells, basophils, and dendritic cells. By lowering free IgE levels and downregulating FcεRI expression, omalizumab reduces IgE-mediated cell activation and subsequent release of inflammatory mediators. Originally approved for the treatment of allergic asthma and chronic spontaneous urticaria, omalizumab has also been explored as a potential therapy for AD, especially in patients with high serum IgE levels or coexisting allergic diseases. In AD, mast cell activation via the IgE-FcεRI pathway contributes to the release of pro-inflammatory cytokines and pruritogenic mediators, perpetuating inflammation and itch. By blocking this pathway, omalizumab may help reduce disease severity. Clinical studies and case reports have shown mixed but generally positive results. Some patients with moderate-to-severe AD experienced improvements in skin lesions, pruritus, and quality of life after omalizumab treatment, particularly those with extrinsic (IgE-associated) AD.

Bruton's tyrosine kinase (BTK) is a critical signaling molecule downstream of FcεRI and the B-cell receptor. BTK inhibitors, such as rilzabrutinib and remibrutinib, have recently emerged as potential therapeutic agents for conditions with mast cell involvement, including chronic spontaneous urticaria and atopic dermatitis (40). By inhibiting BTK, these drugs can reduce mast cell activation and subsequent release of pro-inflammatory mediators, thereby alleviating pruritus, inflammation, and other AD-related symptoms. In this Phase 2b trial of chronic spontaneous urticaria (CSU) patients, the oral Bruton tyrosine kinase (BTK) inhibitor remibrutinib demonstrated rapid efficacy, with all dose groups showing significant reductions in Weekly Urticaria Activity Score (UAS7) by Week 4 (−14.7 to −20.0 vs. −5.4 for placebo, *P* < 0.0001) and sustained improvement through Week 12. The drug exhibited a favorable safety profile, with predominantly mild-to-moderate adverse events and no dose-dependent toxicity, positioning it as a promising oral therapy for H1-antihistamine-refractory CSU (60). In this CSU trial, rilzabrutinib (1,200 mg/day) significantly improved itch (−9.21 vs. placebo −5.77) and hives (−16.89 vs. −10.14) by Week 12 (both *p* = 0.02), with effects starting at Week 1. The oral BTK inhibitor showed favorable safety (mainly diarrhea/nausea) and reduced key inflammatory markers (61). The inclusion of BTK inhibitors highlights the expanding landscape of targeted therapies that modulate mast cell function in AD.

However, efficacy may vary depending on IgE levels, age, and disease phenotype. Omalizumab is generally well-tolerated, with a favorable safety profile. While it is not yet approved specifically for AD, it may be considered as an off-label option for select patients, particularly those with allergic sensitization or refractory disease unresponsive to conventional treatments. Ongoing research and clinical trials continue to evaluate its effectiveness and identify biomarkers that predict therapeutic response in AD.

### 8.2 Targeting type 2 cytokines

In recent years, monoclonal antibodies targeting type 2 inflammation have revolutionized the treatment landscape for AD, particularly in moderate-to-severe cases. Among the most prominent biologic therapies are dupilumab and nemolizumab, both of which interfere with key cytokines driving type 2 immune responses that are central to AD pathogenesis.

Dupilumab is a fully human monoclonal antibody that blocks the shared alpha subunit of the IL-4 and IL-13 receptors (IL-4Rα). IL-4 and IL-13 are critical cytokines in the type 2 inflammatory response and play major roles in promoting IgE class switching, eosinophil recruitment, pruritus, and impairment of skin barrier function. By inhibiting IL-4/IL-13 signaling, dupilumab effectively reduces the inflammation and barrier dysfunction characteristic of AD (62, 63). Dupilumab has been approved by the FDA and other regulatory agencies for the treatment of moderate-to-severe AD in adults and children (aged 6 months and older). In large-scale clinical trials, dupilumab significantly improved eczema severity scores, reduced itch, improved sleep and quality of life, and decreased the use of topical corticosteroids. Improvements can be seen within weeks of initiation and are sustained with long-term treatment. Dupilumab reduces Th2 cell activation, lowers serum IgE levels, and promotes skin barrier restoration by normalizing keratinocyte differentiation and decreasing expression of inflammatory genes in lesional skin.

Tralokinumab and lebrikizumab are humanized monoclonal antibodies developed for the treatment of moderate-to-severe atopic dermatitis (AD), a chronic inflammatory skin disease (64, 65). Both therapies specifically target interleukin-13 (IL-13). IL-13 contributes to skin inflammation, pruritus, epidermal barrier dysfunction, and reduced antimicrobial peptide expression.

Tralokinumab binds directly to IL-13 with high specificity, preventing it from interacting with both IL-13 receptor subunits—IL-13Rα1 and IL-13Rα2—thereby blocking downstream signaling pathways involved in inflammation and immune dysregulation (65). Lebrikizumab also targets IL-13 but in a slightly different manner. It binds to IL-13 and inhibits its interaction specifically with the IL-13Rα1 receptor, without blocking IL-13's binding to IL-13Rα2. This selective inhibition blocks the IL-4Rα/IL-13Rα1 receptor complex formation, which is critical for activating STAT6 signaling, a pathway driving many features of AD.

Nemolizumab is a humanized monoclonal antibody that targets the IL-31RA, thereby blocking the activity of IL-31, a cytokine predominantly associated with pruritus in AD and other chronic skin disorders. IL-31 is produced by activated Th2 cells and mast cells and directly stimulates cutaneous sensory nerves, contributing to the intense itch that characterizes AD. In phase 2 and 3 trials, patients treated with nemolizumab experienced a rapid and sustained reduction in itch severity, often within days of starting treatment (66). By selectively inhibiting IL-31 signaling, nemolizumab interrupts the itch-scratch cycle—a critical driver of chronic inflammation and skin barrier disruption in AD (67). Unlike dupilumab, nemolizumab does not target broader type 2 inflammation, making it a more focused option for symptom control, particularly pruritus. Nemolizumab has been well-tolerated in clinical studies, with adverse events including mild injection site reactions, upper respiratory tract infections, and occasional worsening of AD in a small subset of patients (68). Long-term safety data are still being collected.

Both dupilumab and nemolizumab represent major advances in the treatment of AD by targeting central mediators of type 2 inflammation and pruritus. Dupilumab offers broad anti-inflammatory effects by inhibiting IL-4 and IL-13, leading to improvements in both skin inflammation and barrier function. Nemolizumab, by targeting IL-31, provides powerful and rapid itch relief, addressing one of the most burdensome symptoms of AD. As research continues, these therapies may be used in combination with other targeted agents or personalized based on patient-specific immune profiles, ushering in a new era of precision medicine in dermatology.

### 8.3 Anti-KIT monoclonal antibodies

Anti-KIT monoclonal antibodies are targeted therapies designed to bind to the KIT receptor (CD117), a tyrosine kinase receptor critical for mast cell survival, proliferation, and activation. Aberrant KIT signaling, often due to gain-of-function mutations (such as D816V), is implicated in various diseases (69), including systemic mastocytosis and gastrointestinal stromal tumors (GIST).

One example is avapritinib, a selective KIT inhibitor effective against the D816V mutation, approved for advanced systemic mastocytosis (70). Another agent, imatinib, was the first KIT-targeting drug, used primarily for GISTs with KIT exon 11 mutations but is ineffective against D816V. Monoclonal antibodies such as KRN7000 and SR1 are under investigation for their potential to block KIT activation or mediate antibody-dependent cellular cytotoxicity (ADCC). These antibodies can reduce mast cell burden and inhibit disease progression by interfering with KIT signaling or promoting immune-mediated clearance of abnormal cells. In clinical settings, anti-KIT therapies are used to manage cancers and mast cell disorders, especially in patients with KIT-driven pathologies (71). Continued research is expanding their application to other KIT-expressing malignancies and allergic diseases.

### 8.4 Mast cell stabilizers

Mast cell stabilizers are a class of medications that work by preventing mast cell degranulation, thereby inhibiting the release of histamine, proteases, and other pro-inflammatory mediators that contribute to allergic reactions and inflammation. These drugs are particularly useful in conditions where mast cell activation plays a central role, such as in AD.

The most commonly used mast cell stabilizer in clinical practice is cromolyn sodium, which is typically available in topical, oral, or inhaled forms (72). Cromolyn works by stabilizing the mast cell membrane, preventing the release of preformed mediators in response to various triggers, including allergens, irritants, and physical stimuli. By doing so, cromolyn can reduce inflammation, itching, and the severity of AD flare-ups.

In AD, mast cells contribute to disease pathology through IgE-dependent activation (via FcεRI) and non-IgE-dependent pathways (e.g., MRGPRX2). These cells release histamine, cytokines, and proteases that promote pruritus, skin barrier dysfunction, and chronic inflammation. By stabilizing mast cells, cromolyn helps to reduce these harmful effects. Although mast cell stabilizers like cromolyn are not first-line treatments for AD, they can be helpful in managing mild to moderate cases or in combination with other therapies. They may be especially beneficial in patients with symptoms related to mast cell activation, such as intense itching and localized inflammation (73). Mast cell stabilizers primarily act on certain non-IgE-dependent mechanisms, particularly highlighting the significance of MRGPRX2 in atopic dermatitis (74, 75). They are often used in conjunction with topical corticosteroids, antihistamines, or other immunomodulatory agents to provide a more comprehensive treatment approach. Mast cell stabilizers are generally well-tolerated with minimal systemic side effects. However, they may be less effective than newer biologic therapies (e.g., dupilumab) and are typically considered second- or third-line options in the management of AD.

### 8.5 Histamine receptor antagonists

Histamine receptor antagonists are medications that block histamine receptors, particularly H1 receptors, to alleviate the symptoms of allergic reactions, including pruritus, which is a hallmark of AD. Histamine is a key mediator released from mast cells during allergic inflammation, and its binding to H1 receptors on sensory nerve endings and other cells contributes significantly to the sensation of itch (76).

H1 receptor antagonists, commonly known as antihistamines, work by blocking the action of histamine on H1 receptors. This reduces the intensity of itching and helps manage other allergic symptoms, such as redness and swelling, in AD (77). Some examples of commonly used H1 antihistamines include cetirizine, loratadine, and fexofenadine.

The highly selective histamine H4 receptor, the antagonist ZPL-3893787, was demonstrated in a phase II clinical trial in AD. It can significantly improve the severity of the patient's disease, and after 8 weeks of treatment. The patient's eczema area and severity index with a 50% improvement and SCORAD with a 41% improvement from baseline (78).

In AD, pruritus is often driven by histamine release from activated mast cells, and antihistamines can be effective in providing symptomatic relief. By reducing histamine-induced itch, these drugs help to alleviate one of the most distressing symptoms of AD, thus improving the patient's quality of life. Antihistamines are typically used as part of a broader treatment strategy for AD, often alongside topical corticosteroids or other immunomodulatory therapies (79). They are most effective for mild-to-moderate pruritus and may be used in combination with topical treatments to reduce the need for oral corticosteroids and other systemic immunosuppressants. Sedating antihistamines (e.g., diphenhydramine) may be used at night to help improve sleep quality in patients suffering from severe itching.

While antihistamines are generally well-tolerated, they can cause drowsiness, particularly the first-generation sedating antihistamines. The efficacy of antihistamines in treating the underlying inflammation of AD is limited, so they are generally not used as monotherapy for disease control (80). They are most effective when combined with other treatments that address the root causes of inflammation and skin barrier dysfunction in AD.

### 8.6 JAK inhibitors

JAK inhibitors are a class of oral medications that target the Janus kinase (JAK) family of enzymes, which play a critical role in the signaling pathways of various cytokines involved in inflammation (81). These include cytokines such as IL-4, IL-13, and IL-31, which are central to the type 2 inflammatory response in AD. JAK inhibitors work by blocking the JAK-STAT (signal transducer and activator of transcription) signaling pathway, preventing the activation of downstream genes that lead to inflammation, immune cell activation, and itch. By inhibiting JAK enzymes (JAK1, JAK2, JAK3, and Tyk2), these drugs reduce the activity of pro-inflammatory cytokines and alleviate symptoms such as itching, redness, and swelling, which are characteristic of AD (82).

Several JAK inhibitors have been investigated or approved for the treatment of moderate-to-severe AD, including tofacitinib, upadacitinib, and abrocitinib. These medications are particularly useful in patients who do not respond well to topical therapies or biologics like dupilumab. Clinical trials have demonstrated significant improvements in the Eczema Area and Severity Index (EASI), reduction in pruritus, and overall skin clearance (83). While JAK inhibitors are effective in controlling AD, they are associated with potential side effects, including increased risk of infections, blood clots, and changes in blood cell counts. Long-term safety data are still being collected. These inhibitors are generally used when other treatments, such as topical corticosteroids or biologics, are insufficient or not tolerated. They offer a valuable option for patients with refractory or severe AD (84). In summary, JAK inhibitors represent an important treatment option for managing moderate-to-severe AD, especially for patients who require systemic therapy or have not responded to other treatments.

### 8.7 Inhibition of MRGPRX2/B2

Inhibition of non-IgE pathways, particularly targeting the MRGPRX2 and MRGPRB2 receptors, represents a novel approach in the treatment of AD. These receptors are part of a family of GPCRs expressed on mast cells and other immune cells, and they are involved in mediating non-IgE-dependent activation of mast cells. In AD, mast cells are activated not only through the classical IgE-dependent pathway (via FcεRI) but also through these non-IgE pathways. MRGPRX2 and MRGPRB2 receptors are activated by various ligands, including neuropeptides, small molecules, and other triggers, leading to mast cell degranulation and the release of pro-inflammatory mediators like histamine, cytokines, and proteases. This non-IgE-mediated activation can contribute significantly to the chronic inflammation, pruritus, and skin barrier dysfunction observed in AD.

Targeting MRGPRX2 and MRGPRB2 provides a potential strategy for modulating mast cell activity in patients with AD who may not respond to traditional IgE-targeted therapies. Inhibitors of these receptors are currently under investigation in preclinical studies and early-phase clinical trials. The aim is to block mast cell degranulation and reduce the inflammatory and pruritic symptoms of AD without the need for IgE-specific therapies, offering a new option for patients with IgE-independent AD or those who do not respond to standard treatments. While targeting non-IgE pathways holds promise, the clinical development of MRGPRX2 and MRGPRB2 inhibitors is still in its early stages. As these receptors are also involved in other physiological processes, careful evaluation of safety and potential off-target effects is essential. Nonetheless, this approach provides a novel avenue for AD treatment, particularly for patients with complex or refractory disease.

### 8.8 Anti-Siglec-8 monoclonal antibodies

Anti-Siglec-8 monoclonal antibodies are a novel class of therapeutics targeting Siglec-8, a cell surface receptor selectively expressed on eosinophils, mast cells, and basophils. Siglec-8 is an inhibitory receptor that, when engaged, induces apoptosis in eosinophils and inhibits mediator release from mast cells, making it a promising target for treating allergic and eosinophilic disorders (85).

Lirentelimab (AK002) is a humanized IgG1 monoclonal antibody targeting Siglec-8. It has shown potent effects in depleting eosinophils and suppressing mast cell activity through antibody-dependent cellular cytotoxicity (ADCC) and direct receptor signaling (86). Lirentelimab has demonstrated clinical efficacy in diseases such as eosinophilic gastritis, eosinophilic esophagitis, and chronic urticaria in early-phase trials (87). By selectively targeting Siglec-8-expressing cells, anti-Siglec-8 antibodies offer a focused approach with potentially fewer systemic side effects compared to broad immunosuppressants. Their ability to reduce inflammation and symptom burden makes them promising for treating a range of eosinophil- and mast cell-associated conditions. Ongoing clinical trials are evaluating their safety, efficacy, and long-term outcomes, with potential expansion into other allergic and inflammatory diseases.

## 9 Conclusion

AD is a chronic inflammatory skin disease with a complex mechanism of action. Mast cells are mainly activated through the IgE- and mx pathways, thereby degranulation and releasing proteases and inflammatory factors to promote the pathogenesis of AD. In addition, mast cells promote the pruritus symptoms of AD through the neuro-immune mechanism. In view of the important role of mast cells in AD, a variety of drugs targeting mast cells and the contents they release have been developed for the treatment of AD, especially the application of biological agents, and have achieved good results. It is expected that more mechanisms and treatment methods targeting mast cells will emerge in the future, further improving the therapeutic effect and prognosis of AD and mast cell-related diseases.
